# Pulmonary vein reconnection patterns after pentaspline pulsed field ablation for atrial fibrillation

**DOI:** 10.1093/europace/euag172

**Published:** 2026-07-02

**Authors:** Alexander Lucius, Stephan Kische, Stefan Mueller

**Affiliations:** Vivantes Klinikum im Friedrichshain, Department of Cardiology, Landsberger Allee 49, Berlin 10249, Germany; Vivantes Klinikum im Friedrichshain, Department of Cardiology, Landsberger Allee 49, Berlin 10249, Germany; Vivantes Klinikum im Friedrichshain, Department of Cardiology, Landsberger Allee 49, Berlin 10249, Germany

Pulsed-field ablation (PFA) has emerged as a non-thermal alternative in interventional atrial fibrillation (AF) therapy with myocardial selectivity and encouraging safety and efficacy profiles for patients undergoing pulmonary vein isolation (PVI). While recent studies suggest favourable lesion durability^1–3^, the anatomical distribution of reconnection after PFA remains incompletely characterized, particularly for pentaspline catheter systems. Previous analyses using circular over-the-wire PFA catheters have demonstrated that reconnection does not occur randomly but rather clusters in specific anatomical regions. These observations suggest that lesion durability is influenced by catheter geometry, lesion deployment strategy, and tissue contact. The present study therefore aimed to characterize pulmonary vein reconnection patterns after index pentaspline PFA (Farapulse, Boston Scientific, MA, USA) PVI in a real-world cohort.

This retrospective, observational, single-centre study included 258 consecutive patients undergoing first-time catheter ablation for both paroxysmal (pAF) and persistent (persAF) using the pentaspline PFA system Farapulse. The initial procedure was conducted with four PFA catheter applications in ‘basket’ configuration and four in ‘flower’ configuration per vein as per manufacturer’s protocol. Additional applications like the ‘olive’ configuration^4^ were used at operator’s discretion. After a 3 month blanking period, patients with documented arrhythmia recurrence (pAF, persAF, or atrial tachycardia lasting >30 s) during follow-up at 3, 6, and 12 months with 3-day Holter ECG recordings (or cardiac implantable electronic devices if present) were considered for repeat procedure. All repeat procedures were performed using a 3D mapping system (Opal HDx, Boston Scientific, MA, USA or CARTO^TM^ 3, Biosense Webster Inc., Irvine, CA, USA). The primary endpoint was the number of reconnected PVs as well as the exact lesion gap location.

A total of 258 consecutive patients (55.8% male) with paroxysmal (34.8%) and persistent (65.2%) AF were included in the analysis. The mean age of patients was 68.9 ± 11.6 years. Major adverse events during or after index PVI procedure were observed as follows: pericardial effusion 2/258 (0.78%), transient cerebral deficit 2/258 (0.78%), vascular access site complication 4/258 (1.54%).

After the index procedure between 29 January 2024 and 29 August 2025, a total of 59/258 patients (22.9%) underwent repeat ablation before 15 April 2026 for pAF (14/59, 23.7%), persAF (21/59; 35.6%), as well as atrial tachycardia (24/59; 40.7%). The mean time to repeat ablation was 266.0 ± 107 days. 51 patients (19.8%) underwent repeat ablation within 1 year of the index procedure. No major adverse events were observed during and after the repeat ablation procedure. Of 236 pulmonary veins assessed, 195 (82.6%) remained durably isolated, whereas 41 (17.4%) demonstrated reconnection. Patients with persistent AF at index presentation comprised 66.7% of the cohort with non-durable PVI. Vein-specific durability rates were 71.2% (42/59) for left superior pulmonary vein (LSPV) and 86.4% (each 8/59) for left inferior pulmonary vein, right superior pulmonary vein (RSPV), and right inferior pulmonary vein, respectively. Durable isolation of all pulmonary veins per patient was present in 20 cases (33.9%). With regard to anatomical lesion gap distribution, reconnection occurred predominantly in the posterior–superior segment of the LSPV/roof junction, the anterior part of the RSPV, and in both carina regions (predominantly anterior) (*Figure 1*). Patients without PV or carina reconnection received a higher number of PFA applications during the index procedure than patients with subsequent reconnection (45.2 ± 9.3 vs. 38.1 ± 7.8 applications, *P* = 0.006). Procedure duration (66.1 ± 21.1 vs. 62.3 ± 22.0 min, *P* = 0.53) and fluoroscopy time (11.1 ± 3.3 vs. 10.5 ± 3.6 min, *P* = 0.51) did not differ between patients with durable lesion sets and those demonstrating pulmonary vein or carina reconnection at repeat ablation.

**Figure 1:**
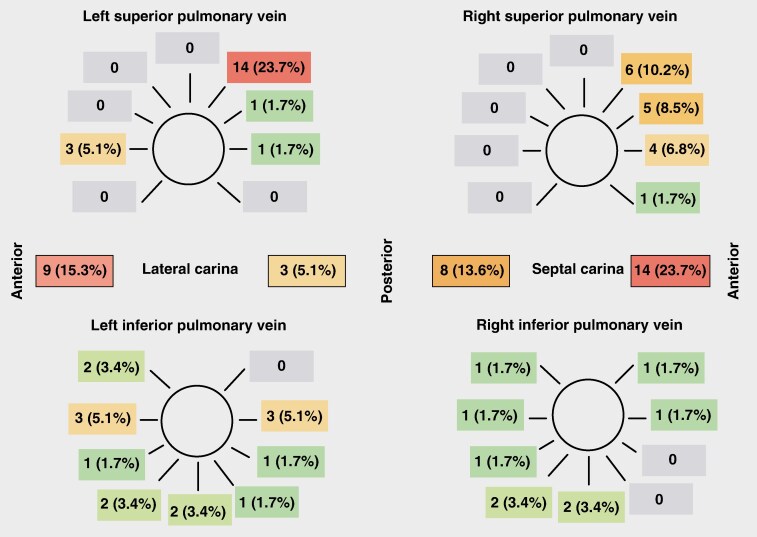
Segmental distribution of pulmonary vein and carina reconnection sites in 59 patients undergoing repeat ablation after index pentaspline pulsed field ablation. Values are presented as the number and percentage of patients with reconnection at the respective anatomical location, using all repeat procedures as the denominator (*n* = 59). Multiple reconnection sites per pulmonary vein or carina were possible. LIPV, left inferior pulmonary vein; LSPV, left superior pulmonary vein; RIPV, right inferior pulmonary vein; RSPV, right superior pulmonary vein.

This study summarizes repeat ablation outcomes in patients who developed documented atrial arrhythmias after undergoing ablation with the Farapulse PFA catheter. So far, detailed description of lesion gap distribution for this first generation PFA catheter is limited.

In the studied cohort, 33.9% of patients undergoing repeat ablation showed persistent isolation of all PVs. Reconnection of at least one PV or the carina region was evident in 66.1%. This is comparable with the recent publication of other PFA systems^5^ as well as thermal ablation systems.^6–8^ The dominant sites of lesion failure were the carina regions and the posterior–superior junction of the LSPV and the atrial roof as well as the anterior part of the RSPV. As in previous studies, we did find more reconnections at the superior veins, but in contrast more often located on the lateral veins.^9,10^ The circular catheter design might play a role in reconnection rates observed in the carina. As for the LSPV/roof junction reconnection, a suboptimal catheter/tissue contact is possible, thereby further highlighting the importance of adequate tissue contact for durable lesion formation. The observed patterns also show similarities to reconnection after cryoballoon ablation, where carina regions and superior pulmonary vein segments are recognized sites of lesion failure.^8,7^ This convergence across different energy sources indicates that geometric factors and anatomical accessibility remain key determinants of durable PVI, regardless of the ablation technology used. Therefore, after preliminary evaluation of these results, we changed our workflow by applying two extra PFA applications in ‘flower’ configuration to each carina, positioning the catheter slightly more roofwards at the LSPV, and applying additional anterior torque during RSPV ablation. The future use of the FaraView integrated 3D mapping system with ContactSense technology (Boston Scientific, MA, USA) might also serve in overcoming this caveat.

Reconnection patterns were assessed only in patients undergoing repeat procedures and therefore reflect a selected subgroup, not the true PV reconnection rate and location. Follow-up time and the number of patients included are still limited, and they were only evaluated retrospectively and in a single centre. The proposed implications for lesion-set optimization are hypothesis generating and require prospective validation.

Pulmonary vein reconnection after pentaspline PFA occurs in reproducible anatomical patterns, predominantly involving the carina, the LSPV/roof junction, and the anterior RSPV. These findings highlight potentially modifiable limitations of current lesion deployment strategies.

## Data Availability

The data underlying this article will be shared on reasonable request to the corresponding author.
